# Effects of negative emotions and information perceived value on residents' risk perception during the COVID-19 pandemic: An empirical survey from China

**DOI:** 10.3389/fpubh.2023.980880

**Published:** 2023-02-20

**Authors:** Chaoyi Chen, Xiaodong Sang, Ruijun Wu, Zhanchun Feng, Chengxu Long, Yisheng Ye, Ziqi Yan, Can Sun, Lu Ji, Shangfeng Tang

**Affiliations:** ^1^School of Medicine and Health Management, Tongji Medical College of Huazhong University of Science and Technology, Wuhan, China; ^2^Division of Strategy and Policy, China Biotechnology Development Center, Beijing, China

**Keywords:** risk perception, negative emotion, information perceived value, COVID-19 pandemic, psychology

## Abstract

**Background:**

The COVID-19 pandemic has spread rapidly and heavily hit the globe, and the mutation and transmission speed of the coronavirus have accelerated so that the world is still in danger. Thus, this study aims to investigate the participants' risk perception and explore the associations of risk perception of COVID-19 with negative emotions, information value perception and other related dimensions.

**Methods:**

A cross-sectional, population-based online survey was conducted from April 4 to 15, 2020, in China. A total of 3,552 participants were included in this study. A descriptive measure of demographic information was used in this study. Multiple regression models and moderating effect analysis were used to estimate the effect of potential associations of risk perceptions.

**Results:**

Those who showed negative emotions (depressed, helplessness, loneliness) and perceived video information in social media to be useful were positively correlated with risk perception, whereas individuals who perceived experts' advice to be useful, shared risk information with friends and thought that their community made adequate emergency preparation reported lower risk perception. The moderating effect of information perceived value (β = 0.020, *p* < 0.001) on the relationship between negative emotion and perception of risk was significant.

**Conclusions:**

Individual differences in risk cognition during the COVID-19 pandemic were observed in subgroups of age level. Furthermore, the role of negative emotional states, the perceived usefulness of risk information and the sense of security also contributed to improving the public's risk perception. It is crucial for authorities to focus on residents' negative emotions and to clarify misinformation in accessible and effective ways in a timely manner.

## Introduction

The new coronavirus disease (COVID-19) hit the whole world heavily in 2020. The rapid spread of the virus evolved into a global public health emergency, and many countries failed to contain the outbreak so that the world faced prolonged danger. According to the weekly epidemiological report, by the end of August 8, 2022, there have been about 5.81 hundred million confirmed cases of COVID-19, including 6,410,961 deaths (1). The public faced uncertain and excessive risk information due to the high risk, infectivity, and severity of the COVID-19 pandemic. This information poses significant challenges to people's behavioral (e.g., irrational behavior) and psychological resilience (2, 3). Timely and accurate risk transmission helps eliminate people's fear of being infected and stabilize public sentiment. However, social media such as WeChat and Weibo are communication modes of group organization and interaction, which could make public opinion regulation and gatekeeping difficult under the impact of information tsunamis. Given the urgency of the pandemic risk, social media may provide convenient ways for users to receive and share uncertain and inaccurate information much faster *via* social networks (4), especially those targeting heterogeneous risk perceivers (5). When emergencies impinge on the tolerable cognitive schema, people become prone to allostatic overload, which could lead to psychological crisis and irrational behavior (6).

Risk perception acts as a buffer between information exposure and decision-making behavior. It is generally defined as a cognitive judgment of the likelihood of encountering hazards when received risk information is minimal (7, 8). In general, it comprises two components: perceived risk susceptibility and perceived risk severity (9). This is a subjective judgment made by people when characterizing and evaluating hazards, and the evaluation of risks is influenced by numerous individual and societal factors and the exposure of people to external pressures (10). Studies have shown that risk perception could trigger the decision-making process to accept health behaviors, especially in an emergency (11). To eliminate the negative effects of emergency hazards, it is necessary to explore public risk perceptions and the residents' underlying processes (7).

Lerner (12) and Lerner et al. (13) studied the impact of different negative emotions (such as dread, helplessness, and anxiety) on risk perception, and demonstrated that fear makes people exaggerate their assessment of risk, especially, in the absence of risk information, individuals will actively seek information related to risk to reduce their information disadvantage. During crises, information disseminated in a public health emergency is another key determinant of risk perception. Different information channels can either heighten or attenuate risk perception (8). As the social amplification of risk framework states, it is important to understand how people interpret the information and the risk propagation path (14). Residents who relied on unofficial sources, such as Weibo and WeChat, were more likely to exaggerate the risk (5), which then affected health information adoption intention by generating fear (15). The risk people perceived was formed by the synthetic assessments of all kinds of information; thus, information disclosure could amplify an individual's risk perception.

Meanwhile, previous studies have focused on many intervening factors related to risk perception, such as demographic characteristics, trust, social environment, and government (16–18). However, few studies have focused on the perspectives of negative emotional states and perceived value of information. Hence, there is a need to understand both negative emotions and information exposure risk factors influencing effective risk management strategies to the people in communities at risk. More efforts are needed to strengthen trust and communication among the government, social media and vulnerable groups to adjust their risk perception regarding the COVID-19 outbreak. Therefore, this study aims to analyze the factors of individuals' risk perception of COVID-19 at different levels.

## Methods

### Study design

To assess the public's reaction to COVID-19, we used a cross-sectional online survey of citizens in China in early April 2020. Participants were recruited from the eastern, central, and western regions of China by using a directional convenient sampling method. Regarding the severity of the epidemic, two severely affected provinces with the highest number of confirmed patients and one province with the lowest number of patients based on the prevalence of COVID-19 in the early pandemic stage were selected from each region (19). Eight provinces, including Guangdong, Zhejiang, Fujian, Hunan, Hubei, Shanxi, Sichuan, and Gansu, were selected. In each province, provincial capitals and another neighboring city based on the feasibility of conducting the survey were selected, and 60 families from rural and urban areas of each city were selected. Finally, 3,552 individuals were included in this survey. Inclusion criteria were as follows: (a) aged 18 years or older; and (b) the place of residence was the local community during the completion of the survey.

We used a self-designed questionnaire containing 168 questions guided by prior studies and related theories in the literature (7, 20, 21). The questionnaire included structured items about basic demographic variables, such as age, gender, education, marriage, region, and household income. Then, items regarding community emergency preparation, forwarding information and perceived value of risk information were set as independent variables. We assessed community emergency preparation by using a single item: “At the beginning of the epidemic, do you think the community management department or village committee was fully prepared?”. Perceived value of risk information was measured by 9 items: “(1) How helpful is the information a family member has told you? (2) How helpful has the information you have been told by a friend or relative been? (3) How helpful is the information exchanged with others by phone, WeChat and QQ? (4) How much does watching TV program information help you? (5) How much does reading newspaper articles (electronic versions) help you? (6) How much does it help you to follow the articles and opinions of social figures forwarded on WeChat, Weibo and QQ? (7) How much does WeChat, a QQ group or forwarded video information from a circle of friends help you? (8) How helpful is authoritative expert advice? (9) How helpful are official mobile phone messages, calls and voice messages to you?”. The scale's Cronbach's α coefficient was 0.800, and it has good validity (KMO = 0.790).

Negative emotions were measured by using these questions, as follows. (1) Depression was measured by the following question: “Would you say since the beginning of the pandemic you have been feeling depressed?” (2) Would you say since the beginning of the pandemic you have been feeling helpless? (3) Would you say since the beginning of the pandemic you have been feeling loneliness? (22). Participants answered the question using a three-point scale from 1 “none” to “totally agree.” In addition, 6 questions were designed to measure participants' risk perception level of COVID-19 based on the related research conducted in China (22). We made risk perception scale (shortened version) as three dimensions: intuitive perceived sensitivity, perceived severity and cognitive judgments. The Cronbach's α coefficient for this dimension was 0.825.

1. Since the outbreak of COVID-19, I have been afraid of being infected with this disease.
2. Since the outbreak of COVID-19, I have been afraid of dying from this disease.
3. Since the outbreak of COVID-19, I have felt nervous after hearing news about COVID-19.
4. I am worried about being infected with COVID-19, so I have difficulty sleeping.
5. Since the outbreak of COVID-19, it has been difficult for me to stay at home for a long time.
6. When someone mentions COVID-19, my heart beats faster.

Risk perception was set as the outcome variable. It was evaluated with the use of a five-point Likert scale ranging from 1 (strongly disagree) to 5 (totally agree), and all 6 items were summarized from 0 to 30. Higher scores indicate greater risk perception level.

Finally, this study examines the common method biases test by exploratory factor analysis, and the variance explanation rate of all the first factor is 29.34% (< 40%), which means common method deviation is not serious. Next, confirmatory factor analyses were conducted. Model fitting results show that GFI < 0.9, most RMSEA > 0.08. These results verified that common methodological variance was acceptable in this study.

### Research model

**Figure 1:**
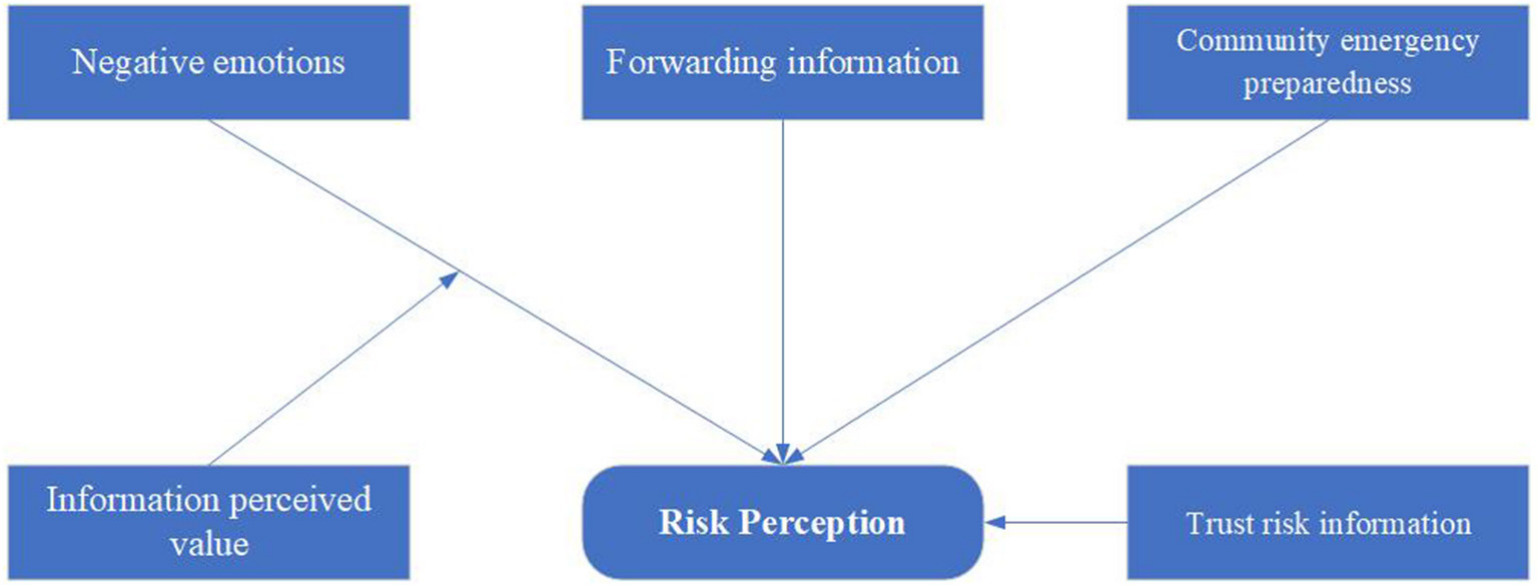
Hypothesized model depicting factors influencing risk perception.

Previous work and studies address a number of crucial determinants that are important for risk perception (19, 23, 24). We hypothesize that residents' negative emotions, forwarding information, community emergency preparedness and trust of risk information may affect risk perception directly. Information perceived value may exert a moderating effect on the relationship between negative emotion and risk perception in the model (Figure 1).

### Data collection

This investigation was conducted online from April 4, 2020, to April 15, 2020. To ensure the randomness of sampling and the reliability of inferences, trained project managers were recruited to coordinate the selected provincial survey and supervise the local investigations. Consenting participants were enrolled and asked to fill out the online questionnaire, which took ~15 min to complete. After receiving the data collected online, we arranged for a dedicated reviewer to be responsible for filtering questionnaires by answering time, content quality, and data format.

### Data analysis

For statistical analysis, we used descriptive methods to summarize data on demographic information. Data were reported as frequencies (n) and percentages (%) for categorical variables among different groups of risk perception. Then, we calculated the means and standard deviations. Multiple regression and moderating effect analysis were used to identify associations between various factors and respondents' risk perception. Statistical analysis in this study was performed by Microsoft Excel and R 3.6.0 software packages. The alpha level was set at *P* < 0.05 for all the analyses.

### Ethical considerations

The study protocols were approved by the Ethics Committee of Tongji Medical College, Huazhong University of Science and Technology (#2020S107). Oral informed consent was obtained from each participant before conducting the online survey.

## Results

### Demographic characteristics of respondents

The average age of the 3,552 participants was 40.67 years old (SD 18.32 years old; age range 10–93 years old). Of these, 52.36% (*n* = 1,860) were women, and 40.68% (*n* = 1445) were unmarried. Older adults (>60 years) accounted for 16.60% (*n* = 590) of the total sample. Furthermore, 51.35% (*n* = 1824) had attained a junior high school degree or higher. Nearly 42.80% of the participants' annual household income was < CNY 100,000. Examination revealed significantly higher mean for risk perception scores in the elderly (>60 years), and the individual who thought their communities made adequate preparation for emergencies (Table 1).

**Table 1:** Demographic characteristics of participants and risk perception score of COVID-19.

**Variables**	**Total (*N* = 3,552)**	**Risk perception score**	***P* value**
	**No (%)**	**Mean** ±**SD**	
**Age (years)**			0.005
< 20	440 (12.39)	17.44 ± 4.23	
21–40	1,351(38.04)	18.05 ± 4.21	
41–60	1,171 (32.97)	17.95 ± 4.34	
>60	590 (16.60)	18.39 ± 4.53	
**Gender**			0.137
Male	1,692 (47.64)	17.88 ± 4.34	
Female	1,860 (52.36)	18.10 ± 4.29	
**Marriage status**			0.247
Unmarried	1,445 (40.68)	17.90 ± 4.19	
Married	2,107 (59.32)	18.07 ± 4.40	
**Household income**			0.350
< CNY 100,000	1,520 (42.79)	18.04 ± 4.33	
CNY 100,000–400,000	1,289 (36.29)	18.07 ± 4.43	
>CNY 400,000	743 (20.92)	17.79 ± 4.08	
**Education**			0.130
≤ 6 years	1,728 (48.65)	18.15 ± 4.47	
7–12 years	1,626 (45.78)	17.87 ± 4.16	
≥13 years	198 (5.57)	17.76 ± 4.16	
**Community preparation**			0.000
Bad	2,092 (58.90)	17.60 ± 4.36	
Fair	793 (22.33)	18.52 ± 4.02	
Good	667 (18.77)	18.61 ± 4.39	

**Figure 2:**
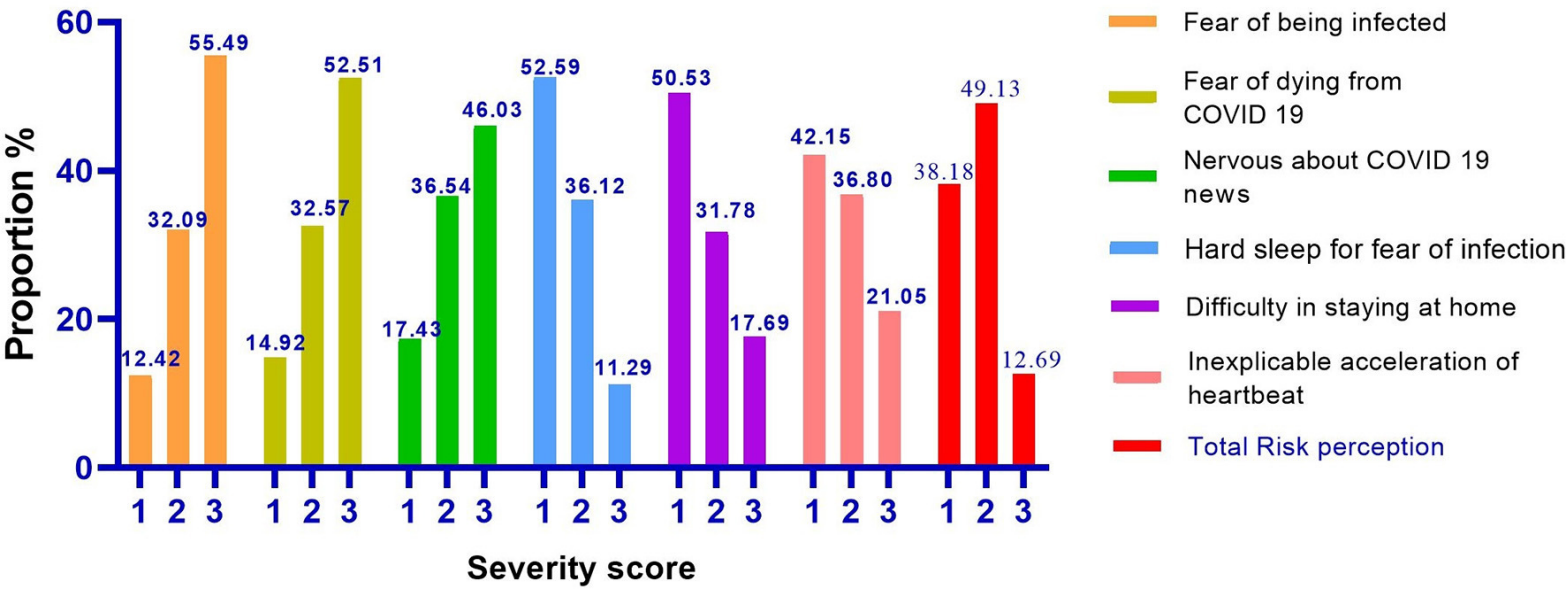
Distribution of the answers related to the participants' risk perception.

The greatest percentage (55.49%) of participants feared being infected; 52.51% feared death from COVID-19; and 46.03% were nervous about reports and news of COVID-19. Respondents did not have difficulty sleeping (52.59%) and had no trouble staying at home (50.53%). When the COVID-19 pandemic was mentioned, 42.15% of respondents did not have symptoms of rapid heartbeat (Figure 2).

### Factors associated with risk perception

Multiple regression analysis showed that participants' risk perception was significantly affected by age, helplessness, depression, loneliness during the pandemic, the usefulness of expert advice, the usefulness of social media video, community's emergency preparation and information forwarding behavior. These variables accounted for 56.33% of the total variance (Table 2).

**Table 2:** Multiple regression results of risk perception.

**Variables**	**β**	**SE**	**t**	***p***
**Intercept**	17.59	0.72	24.44	0.000^***^
**Gender (Ref: Male)**	0.11	0.14	0.77	0.443
**Age (years, Ref:** ≤ **20)**				
21–40	0.45	0.24	1.89	0.059
41–59	0.70	0.29	2.41	0.016^*^
≥60	1.18	0.31	3.87	0.000^***^
**Education level (Ref:** ≤ **6 years)**				
7–12 years	−0.28	0.17	−1.65	0.099
≥13 years	−0.62	0.33	−1.87	0.060
**Household income (Ref:** ≤ **CNY 100 K)**				
CNY 100 K−400 K	0.16	0.16	1.03	0.304
>CNY100 K−400 K	0.03	0.19	0.17	0.865
**Marital status (Ref: Unmarried)**				
Married	−0.08	0.19	−0.43	0.665
**Helplessness (Ref: None)**				
Moderate	1.84	0.35	5.25	0.000^***^
Serious	−0.05	0.21	−0.22	0.822
**Depression (Ref: None)**				
Moderate	0.91	0.18	4.95	0.000^***^
Serious	1.44	0.42	3.39	0.000^***^
**Loneliness (Ref: None)**				
Moderate	0.97	0.18	5.38	0.000^***^
Serious	1.20	0.38	3.13	0.002^**^
**Usefulness of experts' advice (Ref: Useless)**				
A little	−0.45	0.21	−2.13	0.034^*^
Very useful	−0.55	0.19	−2.97	0.003^*^
**Usefulness of social media video (Ref: Useless)**				
A little	0.54	0.18	2.94	0.003^**^
Very useful	0.55	0.19	2.90	0.003^**^
**Forwarded information with friends (Ref: Yes)**				
None	−0.97	0.25	−3.90	0.000^***^
**Community preparation (Ref: Adequate)**				
Moderate	0.75	0.17	4.42	0.000^***^
Inadequate	0.55	0.18	2.95	0.04^*^
**Trust traditional media (Ref: No)**				
Neutral	0.65	0.78	0.83	0.406
Trust	0.55	0.60	0.92	0.356
**Trust friends (Ref: No)**				
Neutral	−0.11	0.70	−1.55	0.122
Trust	−0.96	0.60	−1.59	0.112

^*^p < 0.05, ^**^p < 0.01, ^***^p < 0.001.

Table 3 shows the results of further evaluation of the moderation effects corresponding to the hypothesis mentioned above. The findings identified a significant moderating effect of information perceived value on the relationship between negative emotion and risk perception. The interaction term of information perceived value and negative emotion can positively predict risk perception (β = 0.020, *p* < 0.001).

**Table 3:** Regression coefficients of moderating interaction effects test.

**Variables**	**Model 1**	**Model 2**	**Model 3**	**Model 4**
	β **(*****SE*****)**	β **(*****SE*****)**	β **(*****SE*****)**	β **(*****SE*****)**
**Fixed effects**				
**Intercept**	16.636^**^ (0.404)	12.726^**^ (0.420)	12.330^**^ (0.494)	14.375^**^ (0.870)
**Control variables**				
Gender	0.243 (0.144)	0.109 (0.136)	0.111 (0.136)	0.120 (0.136)
Age	0.017^**^ (0.006)	0.020^**^ (0.005)	0.020^**^ (0.005)	0.020^**^ (0.005)
Education level	−0.027 (0.072)	−0.089 (0.068)	−0.086 (0.068)	−0.085 (0.068)
Marriage	−0.180 (0.186)	−0.066 (0.175)	−0.058 (0.175)	−0.056 (0.175)
**Predictors**				
Forwarded information with friends	−0.827^**^ (0.260)	−1.071^**^ (0.244)	−1.079^**^ (0.244)	−1.100^**^ (0.244)
Community emergency preparation	0.303^**^ (0.049)	0.196^**^ (0.047)	0.198^**^ (0.047)	0.206^*^ (0.047)
Negative emotion		1.000^**^ (0.046)	0.995^**^ (0.046)	0.518^**^ (0.173)
Information perceived value			0.018 (0.012)	−0.071^*^ (0.033)
Negative emotion × Information perceived value				0.020^**^ (−0.007)
***R* ^2^**	0.016	0.130	0.131	0.133
***F***	9.359^**^	75.725^**^	66.580^**^	60.206^**^
**Residual**	4.281	4.025	4.024	4.020

^*^p < 0.01; ^**^p < 0.001.

**Figure 3:**
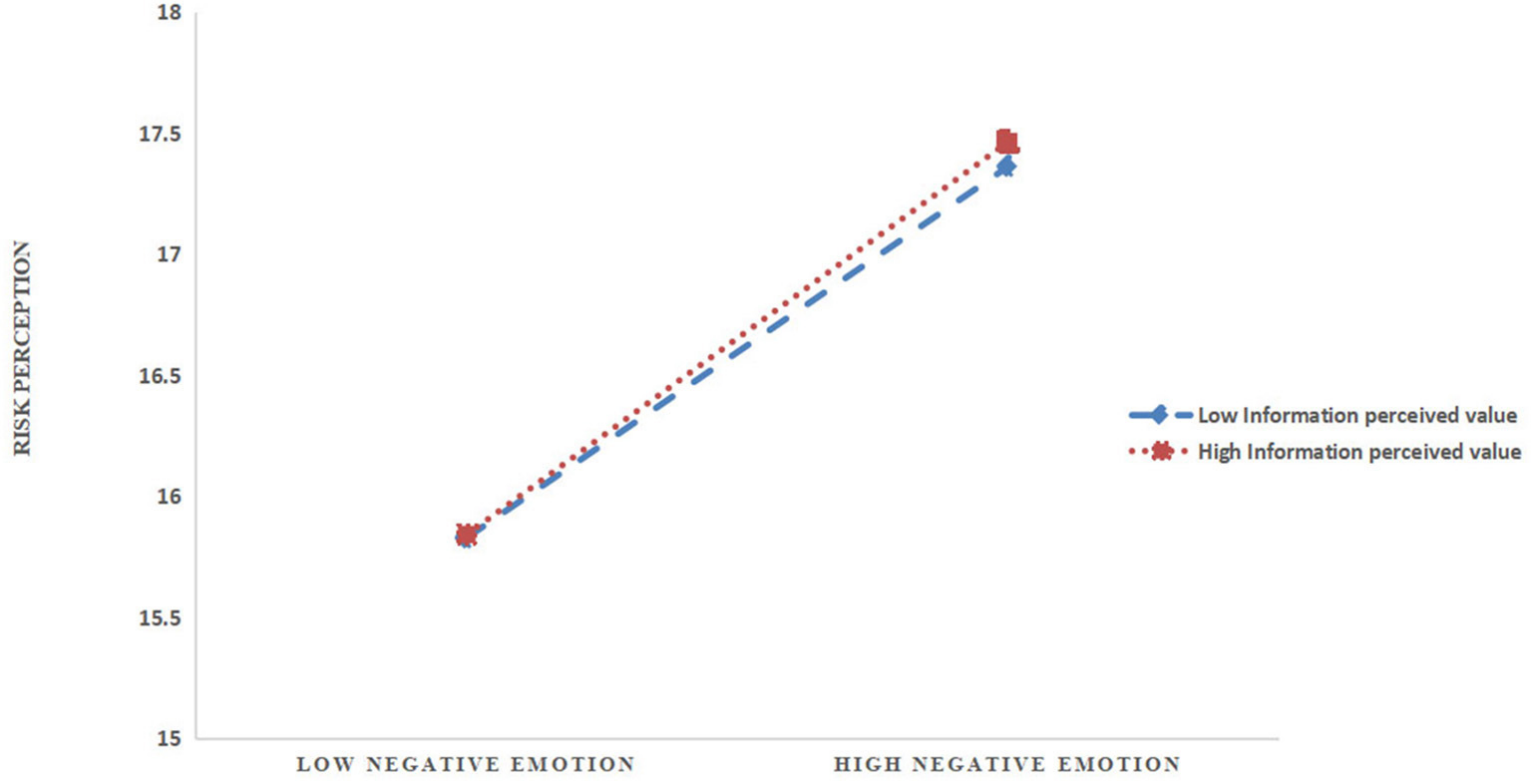
Interactive effects of information perceived value and negative emotion on risk perception.

An interaction plot was generated for visual illustration. Figure 3 indicates that for residents with high value of information perceived, the influence of negative emotion on their risk perception was positive and statistically significant (β = 0.020, *p* < 0.001), and it had a steeper slope, meaning it was even more statistically significant. This finding indicated that the relationship between negative emotion and risk perception would be stronger for those who have higher levels of information perceived value, as compared to those with the lower, but these relationships overall stay positive.

## Discussion

### Main findings of the study

This study indicated that risk perception varied at different ages. Participants who are over 60 years old reported the highest risk perception score in regard to the COVID-19 pandemic; Risk protection awareness of vulnerable and susceptible people and prior emergency experiences may have played a certain role among these participants (25). In addition, we found that factors such as emotion, information perceived value and forwarding behavior were related to an individual's risk perception of COVID-19 among Chinese residents. Moreover, information perceived value exerts a moderating effect on the relationship between negative emotion and risk perception.

This study also demonstrated that adequate community emergency preparedness can provide a vital measure for the government in helping people strengthen self-protection and reduce risk perception. One possible explanation is based on the unique “community grid governance model” in China (26), which is used to build a horizontal network of alliances with other grassroots epidemic prevention sectors (27). Specifically, each community staff member contacts the household to conduct health situation analysis and propagate anti-epidemic, self-protection knowledge and to support residents' interests and public needs (28). Hence, comprehensive community emergency preparedness could raise people's sense of safety.

### The impact of public sentiment and information exposure on risk perception

Another finding of this study was that negative emotions were positively correlated with the perceived likelihood of risks and the perceived severity of the threats. Loneliness, depression, and helplessness were chosen to reflect an individual's negative emotions (psychosocial situation) (29). Some elegant researches by Lerner and Keltner (12, 20) highlighted the mechanism of emotion-specific on risk perception. They predicted and found that negative emotion such as fear and helpless had opposite effects on risk perception. Whereas the helpless people expressed pessimistic risk estimates and they were likely to induce and heighten a pessimistic assessment of perceived severity (30–33). Any perception of risk uncertainty, in turn, influences individuals' emotional response in a hazardous situation. Perceived hazards surrounding COVID-19 can again increase the public's anxiety, depression and fear (34). Furthermore, the spread of the hazard amplifies a strong negative emotional response, which disrupts people's cognition and decreases their belief that they can control negative outcomes (35). In particular, when the individual is under a highly threatening and psychologically stressful condition over a long period of time (36), it increases difficulties in understanding health information and balancing mental resilience protection.

The role of information exposure was positively associated with risk perception. Regression analyses confirmed that in the risk information dimension, participants had higher risk perception when they forwarded risk-related messages with their friends. People who are willing to share risk information usually seek out more media coverage of epidemic-related information in advance. Furthermore, their perceived stress response increases with excessive exposure to risk information (36–38).

Our survey indicated that individuals' perceived value of authoritative information sources has a critical effect on their health risk perception. This is in line with the results of Jian Raymond's and Qingchuan Liu's studies (38, 39). The premise of perceiving the usefulness of information is based on trust; compared with social media, people need to rely more on experts and government agencies' risk communication (40). This highlighted that suggestions from authoritative medical professionals (such as Zhong Nanshan, Li Lanjuan, and Zhang Wenhong) are public health efforts that can help relieve residents' tension and anxiety regarding the epidemic (41). As experts are trustworthy sources and communicate essential protection knowledge to the public, they can help effectively mitigate risk perception (40, 42).

In addition, information perceived value exerts a moderating effect on the relationship between negative emotion and epidemic risk perception. At a high information perceived value, individuals with high negative emotion orientation are relatively more able to perceive the impact of risk (25). People with high perceived value of information rely more on external environment to judge the severity of risks. The original individual and local risk perception in the real world were amplified into the collective and overall risk perception through multiple channels of information dissemination (43). During the epidemic, potential risk and uncertain situations may lead to the public's urgent need for valuable and reliable information, but the volume of discordant and excessive information about COVID-19 makes the concerns over the pandemic seem greater. This is consistent with Mohmmed Salah Hassan's study that demonstrated that the quality of social media contents shaped the individual's perceived susceptibility to a particular public health hazard (44). In addition, this factor might induce a pessimistic assessment of risk information and can heighten feelings of fear and hopelessness with exposure to useless and misleading information. Negative emotional polarization could increase respondents' risk perception.

### Limitation

This study had some limitations. First, many elderly individuals who had no access to the internet were not adequately investigated, and there was a lack of a random sample. Second, we omitted variable bias in this study, and there needs to be a more specific measure (standard questions) of the public's risk perception. Third, a cross-sectional design did not produce very precise or convincing results and made it uncertain. Last, there is a reciprocal cause-effect relationship between risk perception and emotion. It is impossible to determine the exact cause-and-effect relationship between them in this study. Further studies are needed to improve the scientific validity of these findings.

## Conclusions

Perceived risks are important for residents' acceptance of government preventive measures. However, the public's excessive dependence on social media weakens the effect of government trust on public risk perception. It is not conducive to the implementation of prevention measures and self-protective behavior. This study highlighted that individual differences in risk perception were not only related to the age factor. Emotion, perceived usefulness of the information, forwarding behavior and thought community made adequate emergency preparation important. It is crucial for authorities to strengthen the management of new media and guide the release of risk information during the epidemic. In addition, the government should pay attention to the complex negative emotions that threaten residents during the epidemic. Effective health communication and education interventions advocated by public health experts are the most accurate sources of information, which could clarify misinformation and answer public concerns in accessible ways.

## Strength

We conducted a nationwide online survey during the peak of the COVID-19 outbreak in China. The results were a satisfactory reflection of respondents' risk perception status during the crisis situation. This is based on two important dimensions of individual perception analysis, namely, information usefulness and negative emotions. Exploring and analyzing the above factors and their association with risk perception have implications for the adjustment of the public communication and risk prevention strategies adopted by the government.

## Data availability statement

The original contributions presented in the study are included in the article/supplementary material, further inquiries can be directed to the corresponding author.

## Ethics statement

The studies involving human participants were reviewed and approved by Ethics Committee of Tongji Medical College, Huazhong University of Science and Technology. The patients/participants provided their written informed consent to participate in this study.

## Author contributions

CC: conceptualization, data curation, formal analysis, investigation, methodology, software, validation, and writing—original draft. ZF: investigation, project administration, and supervision. ZY: data curation, investigation, and methodology. CS: data curation, investigation, and visualization. ST: conceptualization, investigation, editing subsequent versions of the manuscript, and revising the original manuscript. LJ: support participant recruitment, support data analysis, and interpretation. XS and RW: investigation, writing—review and editing, and supervision. All authors have read and approved the manuscript.
